# Endoscopic ultrasound-guided ileocolostomy using a novel lumen-apposing metal stent for small-bowel obstruction with peritoneal carcinomatosis

**DOI:** 10.1055/a-2228-4691

**Published:** 2024-01-23

**Authors:** Kyong Joo Lee, Se Woo Park, Dong Hee Koh, Jin Lee

**Affiliations:** 1 Division of Gastroenterology, Department of Internal Medicine, Hallym University Dongtan Sacred Heart Hospital, Hallym University College of Medicine, Gyeonggi-do, Korea (the Republic of); 2 Division of Gastroenterology, Department of Internal Medicine, Hallym University Dongtan Sacred Heart Hospital, Hallym University College of Medicine, Hwaseong, Korea (the Republic of)

Peritoneal carcinomatosis is a serious condition stemming primarily from advanced gastrointestinal cancers, which causes significant symptoms such as vomiting due to malignant bowel obstruction (MBO) 1 2 . The prognosis is poor, treatments often lack evidence-based guidelines, and many interventions, including surgical options for related MBO, present challenges 3 . Herein, we present a case of endoscopic ultrasound (EUS)-guided ileocolostomy using a novel lumen-apposing metal stent (LAMS) to treat MBO associated with peritoneal carcinomatosis.

A 52-year-old woman presented to our hospital with frequent vomiting. She had undergone subtotal gastrectomy with Billroth I anastomosis 15 years previously. She had recently been diagnosed with recurrence of peritoneal carcinomatosis. An abdominal computed tomography (CT) scan revealed significant dilatation of the entire small intestine ( Fig. 1 **a** ) and a leading stricture in the distal ileum ( Fig. 1 **b** ). Given the patient’s wishes and her unsuitability for surgery, we first attempted to place an enteral stent using a cap-assisted colonoscope; however, even at the point of the scope’s furthest reach, 30 cm from the ileocecal valve, the ileal stricture could not be accessed.

**Fig. 1:**
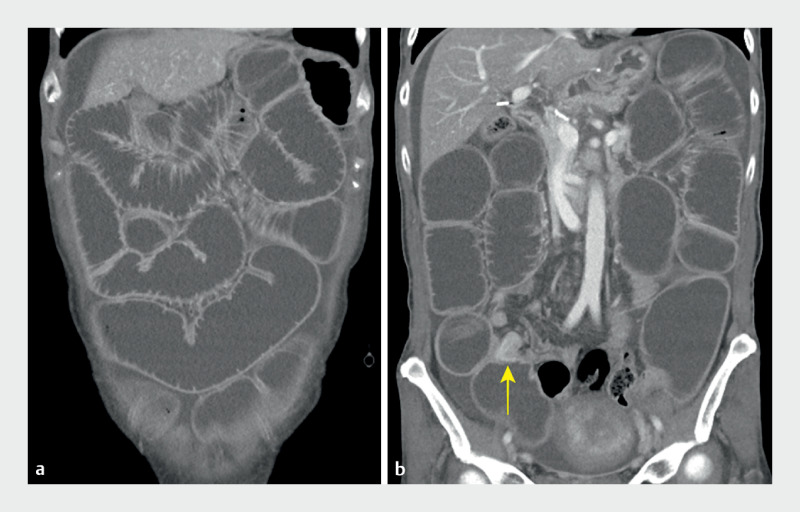
Images from the initial computed tomography scan showing: **a** significant dilatation of the entire small intestine, accompanied by minimal ascites; **b** a pronounced stricture (yellow arrow) resulting from the recurrence of peritoneal dissemination in the distal ileum.

After this unsuccessful attempt, we opted to perform a transluminal EUS-guided ileocolostomy ( Video 1 ). Using a linear echoendoscope (EG-580UT; Fujifilm Medical Systems, Tokyo, Japan), we identified significant dilatation of the ileum. The distal ileum in the pelvic cavity was then punctured from the sigmoid colon using a standard 19-gauge needle (EZ Shot3; Olympus Medical, Tokyo, Japan). Following needle puncture, contrast agent was injected to visualize the distal ileum fluoroscopically. Subsequently, a guidewire was placed and coiled in the distal ileum. Finally, a novel LAMS with an electrocautery-enhanced tip (Niti-S HOT SPAXUS; Taewoong Medical, Gyeonggi-do, South Korea) was inserted and deployed across the ileocolic tract. Upon successful deployment, a substantial volume of liquid fecal material flowed into the sigmoid colon via the LAMS.

Endoscopic ultrasound (EUS)-guided ileocolostomy using a novel lumen-apposing metal stent (LAMS) to treat malignant bowel obstruction.Video 1

Following the intervention, the patientʼs symptoms improved notably, and there were no immediate or delayed adverse events. A subsequent CT scan verified appropriate positioning of the LAMS, connecting the distal ileum and sigmoid colon ( Fig. 2 ). EUS-guided ileocolostomy using this novel LAMS is a viable alternative to surgery for the management of MBO.

**Fig. 2:**
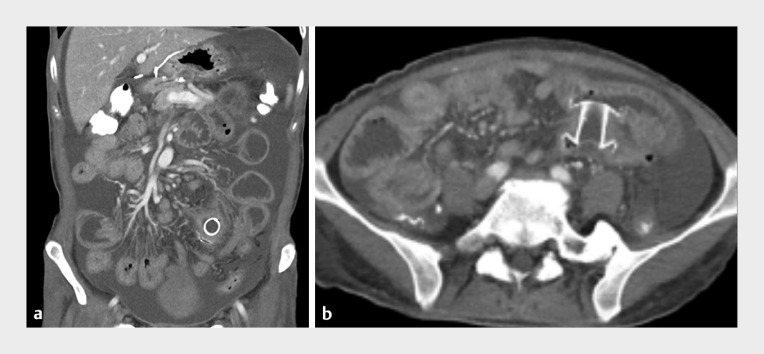
Images from the subsequent computed tomography scan 2 weeks after the endoscopic ultrasound-guided ileocolostomy showing the correctly positioned lumen-apposing metal stent, which has decompressed the entire small intestine.

Endoscopy_UCTN_Code_TTT_1AS_2AZ

## Notes

Endoscopy E-Videos https://eref.thieme.de/e-videos
*E-Videos* is an open access online section of the journal *Endoscopy* , reporting on interesting cases and new techniques in gastroenterological endoscopy. All papers include a high-quality video and are published with a Creative Commons CC-BY license. Endoscopy E-Videos qualify for HINARI discounts and waivers and eligibility is automatically checked during the submission process. We grant 100% waivers to articles whose corresponding authors are based in Group A countries and 50% waivers to those who are based in Group B countries as classified by Research4Life (see: https://www.research4life.org/access/eligibility/ ). This section has its own submission website at https://mc.manuscriptcentral.com/e-videos .
